# Refractory myeloid sarcoma with a FIP1L1-PDGFRA rearrangement detected by clinical high throughput somatic sequencing

**DOI:** 10.1186/s40164-015-0026-x

**Published:** 2015-10-08

**Authors:** Diana Mandelker, Paola Dal Cin, Heather A. Jacene, Philippe Armand, Richard M. Stone, Neal I. Lindeman

**Affiliations:** Department of Pathology, Brigham and Women’s Hospital, Harvard Medical School, 75 Francis St., Boston, MA 02115 USA; Division of Nuclear Medicine, Department of Imaging, Dana-Farber Cancer Institute, Harvard Medical School, 450 Brookline Ave, Boston, MA 02215 USA; Division of Hematological Malignancies, Department of Medical Oncology, Dana-Farber Cancer Institute, Harvard Medical School, 450 Brookline Ave, Boston, MA 02215 USA

**Keywords:** Myeloid sarcoma, FIP1L1-PDGFRA, Somatic sequencing, Next generation sequencing

## Abstract

Next generation sequencing (NGS) is increasingly being used clinically to characterize the molecular alterations found in patients’ tumors. These testing results have the potential to affect clinical care by guiding therapeutic approaches based upon genotype. NGS based testing approaches have a distinct advantage over provider-ordered single gene testing in that they can detect unexpected, yet clinically important genetic changes. Here, we illustrate this principle with the case of a 33-year-old man with myeloid sarcoma that was refractory to six different chemotherapeutic regimens. Our clinical NGS assay detected an unanticipated FIP1L1-PDGFRA rearrangement in his tumor. The patient was immediately placed on Imatinib therapy to which he responded, and remains in remission 10 months after the rearrangement was initially detected.

## Background

As cancer therapeutics increasingly target molecular alterations, testing for somatic changes in cancer is becoming an integral part of pathology evaluations. While these somatic alterations may only be present in a small subset of a given tumor type, patients with these genetic changes often show dramatic responses when treated with targeted agents [1, 2]. Single gene assays evaluating genes such as EGFR in lung adenocarcinoma or BRAF in melanoma are now performed routinely. However, a more comprehensive tumor profiling approach has the advantage of potentially identifying a breadth of actionable genetic alterations [3, 4]. Moreover, these gene panels for somatic testing can identify unexpected genetic changes in cancer types not generally associated with a given somatic alteration.

At the Center for Advanced Molecular Diagnostics (CAMD) at Brigham and Women’s Hospital, a targeted next generation sequencing assay (OncoPanel) is performed in a Clinical Laboratory Improvement Amendments-certified laboratory to detect somatic mutations, copy number variations, and structural variants across 300 cancer-associated genes. For all patients presenting to Dana Farber Cancer Institute who are likely to require systemic therapy, informed consent is obtained, tumor adequacy is assessed, and Oncopanel is run to obtain a somatic profile for the patient’s cancer.

Here, we report a case of a 33-year-old man with myeloid sarcoma that was refractory to six different chemotherapy regimens. His tumor was analyzed using OncoPanel, and a FIP1L1-PDGFRA rearrangement was detected. As hematologic malignancies with this rearrangement are known to be responsive to imatinib therapy, he was placed on imatinib as soon as the rearrangement was reported. He responded rapidly to this course of treatment and currently shows no evidence of residual disease. This case is an example of an unanticipated finding detected through clinical high throughput somatic sequencing that tremendously affected a patient’s care and outcome.

## Case presentation

### Clinical presentation

**Fig. 1:**
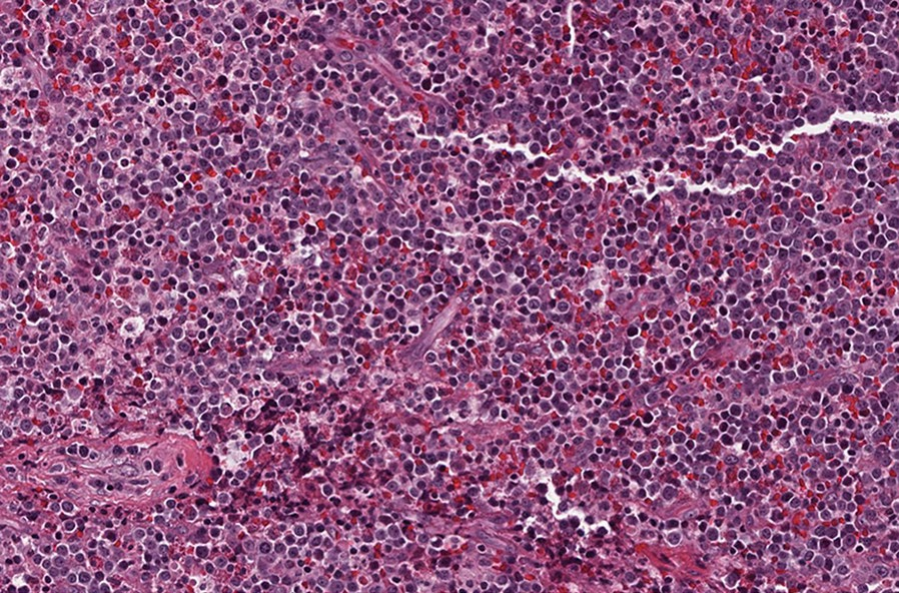
Hematoxylin and eosin stained slide of left neck biopsy. A diffuse proliferation of intermediate to large mononuclear cells with round to irregular nuclei, dispersed chromatin, distinct nucleoli, and small amounts of cytoplasm, consistent with blast forms are seen. Admixed are plasma cells, small lymphocytes, and additional myeloid elements, including abundant eosinophilic forms

**Fig. 2:**
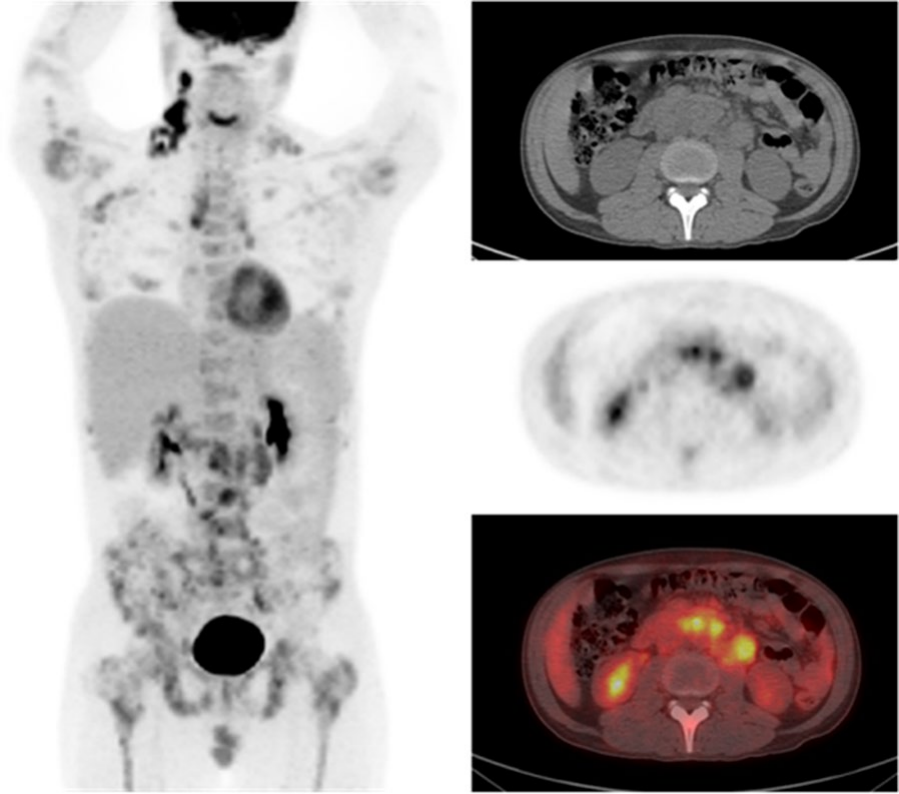
Radiology showing extent of disease burden. PET-CT showing extensive intensely FDG-avid lymphadenopathy above and below the diaphragm and extensive FDG-avid skeletal/marrow disease burden

The patient is a 33-year-old man who was initially diagnosed at an outside hospital with a peripheral T cell lymphoma, based upon a supraclavicular lymph node biopsy. He failed to respond to a regimen of CHOP, and then did not respond to a course of EPOCH. Another lymph node biopsy was performed, and the biopsy showed myeloid sarcoma that was FLT3 ITD and NPM1 mutation negative. He was then transferred to our institution, where a CBC revealed a WBC count of 10.1 K with 9 % lymphocytes, 2 % eosinophils, 3 % basophils, 1 % atypical forms and no circulating blasts. A biopsy of a left neck lymph node was performed and flow cytometry found that 10 % of the cells were phenotypically were positive for CD34, CD45(dim), HLA-DR, CD56, CD34, and myeloid markers CD13, CD33, and CD117, and negative for TdT, CD11b, CD15, and other monocytic, B and T lymphoid markers, consistent with myeloblasts. Surgical pathology sections showed a diffuse proliferation of intermediate to large mononuclear cells with round to irregular nuclei, dispersed chromatin, distinct nucleoli, and small amounts of cytoplasm, consistent with blast forms. Additionally, admixed plasma cells, small lymphocytes and abundant eosinophilic forms were noted (Fig. 1). Cytogenetics showed trisomy 8, and a diagnosis of myeloid sarcoma was rendered. A regimen of 3 days of daunorubicin and 7 days of cytarabine was started, but a follow up PET-CT 4 weeks after initiation of treatment showed progressive disease. He was switched to high dose ARA-C, but again his tumor progressed on PET-CT scan. He then received clofarabine and cytarabine as fifth-line treatment, but his disease continued to progress (Fig. 2), and he was switched to decitabine therapy. His CBC then revealed pancytopenia with 6 % circulating blasts, 6 % atypical forms, and 0 % eosinophils. While the patient was receiving decitabine, the OncoPanel testing of a left neck lymph node was resulted.

### OncoPanel next generation sequencing

**Fig. 3:**
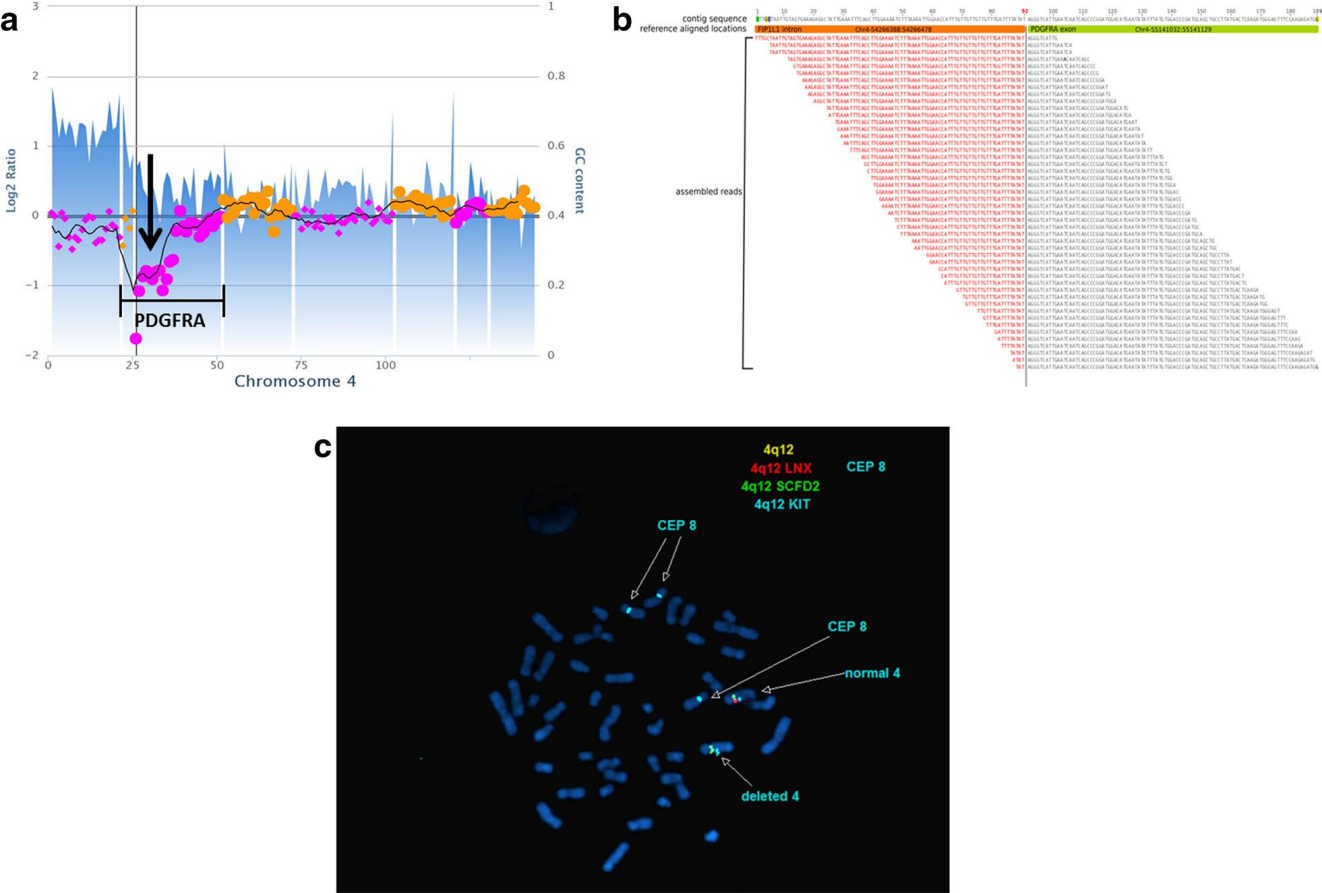
Molecular diagnostics of the patient’s tumor. **a** Copy number assessment of chromosome 4 shows a one copy loss of the 5′ end of PDGFRA. **b** Translocation analysis shows the discordant reads map to intron 10 of FIP1L1 and exon 12 of PDFGRA. **c** Metaphase FISH analysis shows one normal copy of chromosome 4, which retains all 3 FISH probes on 4q (*green* SCFD2 that is centromeric to FIP1L1, *orange* LNX that is located between FIPL1 and PDGFRA, and *blue* KIT that is telomeric to PDGFRA). The other copy of chromosome 4 shows an isolated deletion of LNX with retention of the flanking SCFD2 and KIT probes, indicative of a FIP1L1-PDGFRA rearrangement. Trisomy 8 is also present in these cells, as evidenced by 3 CEP 8 probe signals

The OncoPanel assay is designed to capture and sequence all coding exons of 300 cancer-associated genes and 91 selected introns across 30 genes for rearrangement detection. Sequencing protocols, informatics pipeline, and copy number variation detection were described previously [5]. Targeted next generation sequence analysis by OncoPanel detected 6 heterozygous single nucleotide variants or small insertions/deletions, including KRAS c.38G>A p.G13D, ARID1B c.165_177CCAGCAGCAGCAG>C p.QQQQ68del, EPHA7 c.2042G>A p.C681Y, FANCA c.2712G>C p.Q904H, GATA4 c.124C>A p.P42T, and MSH6 c.359T>C p.I120T. Copy number analysis from Oncopanel confirmed cytogenetic findings that the patient’s myeloid sarcoma contained trisomy 8. Moreover, analysis of chromosome 4 showed a one copy loss of the 5′ end of PDGFRA (Fig. 3a). BreaKmer, the Oncopanel algorithm that uses a ‘kmer’ strategy to assemble misaligned sequence reads for predicting structural rearrangements [6], detected discordant sequencing reads indicative of a FIP1L1-PDGFRA rearrangement (Fig. 3b).

### FISH confirmation

On the basis of the OncoPanel findings, FISH analysis for FIP1L1-PDGFRA fusion was performed on interphase and metaphase nuclei with the Vysis LSI 4q12 Tri-Color Rearrangement Probe Set (Abbott Molecular) that uses three probes (SCFD2, LNX, KIT) on chromosome band 4q12. In this assay, FIP1L1-PDGFRA fusion is indicated by isolated deletion of LNX with retention of the flanking SCFD2 and PDGFRA probes. Only one LNX hybridization signal was observed in 42/100 nuclei (42 %) with retention of the flanking probes, consistent with a FIP1L1-PDGFRA rearrangement (Fig. 3c). Moreover, the cells with a detected FIP1L1-PDGFRA rearrangement also contained trisomy 8, which was a previously demonstrated marker of this patient’s myeloid sarcoma. Trisomy 8 was evidenced by the presence of three signals using he CEP 8 Spectrum Orange DNA Probe Kit (Abbott Molecular), which detects chromosome 8 alpha satellite sequences at 8p11.1-q11.1 was used to interrogate chromosome 8 copy number (Fig. 3c).

### Clinical follow-up

**Fig. 4:**
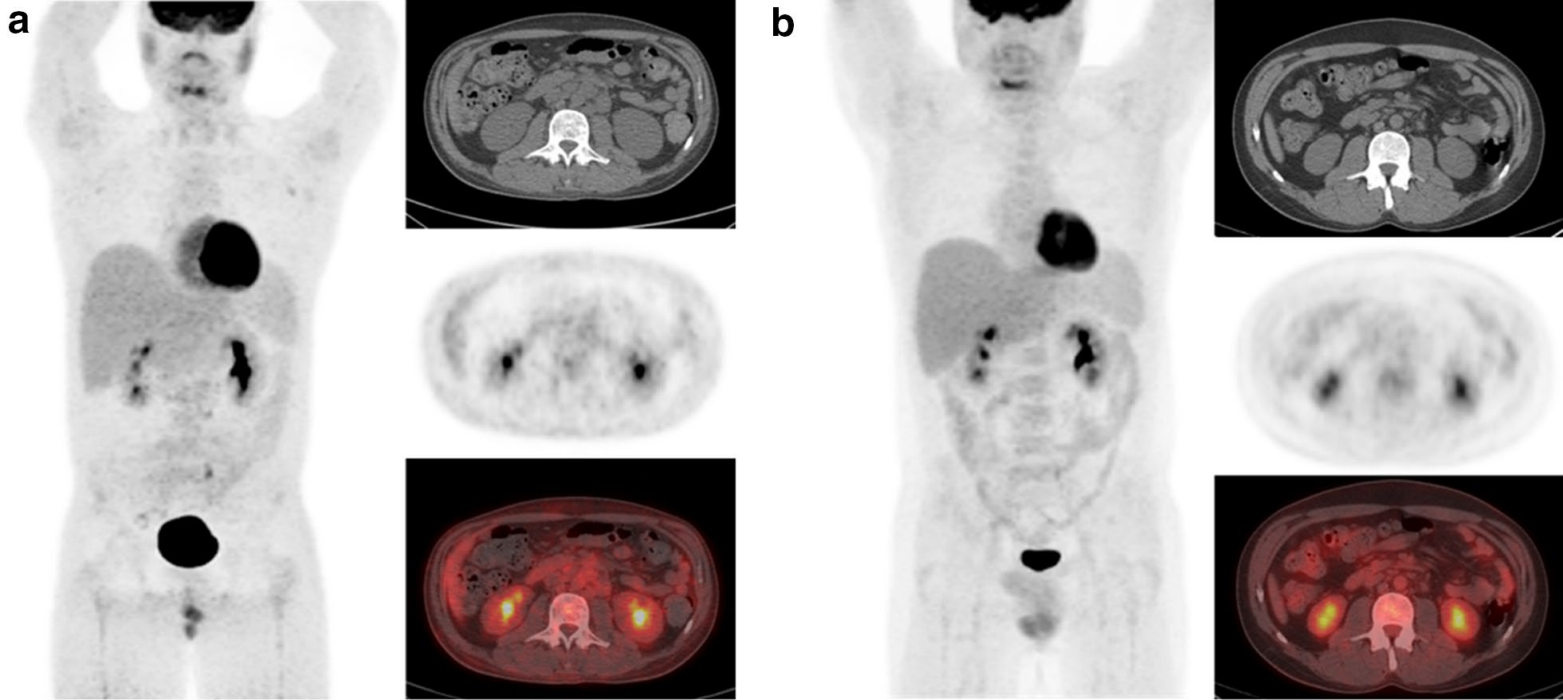
Radiology evaluation of disease burden after imatinib treatment. **a** PET-CT 4 weeks after initiation of imatinib treatment showing marked interval improvement in supradiaphragmatic and infradiaphragmatic lymphadenopathy as well as the skeletal/marrow disease burden. **b** PET-CT 6 months post imatinib treatment and 4 months reduced-intensity conditioning allogeneic stem cell transplantation shows no FDG-avid malignancy

On the same day as the OncoPanel results were reported, the patient was started on 400 mg imatinib daily. Within 1 week of initiating therapy with imatinib, his peripheral blast count decreased from 6 to 0 %, where it has remained. PET-CT performed 4 weeks later showed marked improvement in the supradiaphragmatic and infradiaphragmatic lymphadenopathy as well as the bone marrow disease (Fig. 4a). This treatment response allowed him to undergo a reduced-intensity conditioning allogeneic stem cell transplantation, and he received peripheral blood stem cells from his HLA-matched brother approximately 10 weeks after starting Imatinib. His post-transplantation course was relatively uneventful with mild graft-versus-host disease controlled with topical therapies. He is currently 9 months post-transplant, remains on imatinib 100 mg daily, and has no evidence of disease (Fig. 4b).

## Conclusions

The finding of a FIP1L1-PDGFRA rearrangement in a patient with refractory myeloid sarcoma was an unexpected finding only discovered because of clinical gene panel testing that is performed on all malignancies presenting to our institution. FIP1L1-PDGFRA was first discovered as a recurrent rearrangement in Hypereosinophilic Syndrome in 2003, and response to imatinib was demonstrated [7]. As the spectrum of malignancies associated with this rearrangement expanded, the 2008 WHO classification developed a category for these neoplasms, “Myeloid and lymphoid neoplasms associated with eosinophilia and abnormalities of PDGFRA, PDGFRB, or FGFR1 [8]”. Since our patient did not have peripheral eosinophilia, there was no indication to order specific testing for the FIP1L1-PDGFRA rearrangement. In retrospect, the initial lymph node biopsy showed a variety of cell types admixed with myeloblasts, including plasma cells, small lymphocytes, and abundant eosinophilic forms. However, these findings alone would likely be insufficient to prompt single gene testing for PDGFRA, PDGFRB, or FGFR1.

The FIP1L1-PDGFRA fusion results in a constitutively activated tyrosine kinase and, in hematopoietic cells, results in growth factor-independent growth [7, 9]. The break points within FIP1L1 vary widely, but the break points within the PDGFRA gene are tightly clustered in exon 12, resulting in the disruption of the autoinhibitory juxtamembrane domain and activation of the kinase [10]. On our next generation sequencing panel, the base pair resolution of our translocation assay showed that this patient’s rearrangement occurred between intron 10 of FIP1L1, and exon 12 of PDGFRA. Therefore, the coordinates of the rearrangement predict the disruption of the juxtamembrane domain and kinase activation in this patient.

The diagnosis of acute myeloid leukemia or myeloid sarcoma with a FIP1L1-PDGFRA rearrangement is extremely rare, with only a handful of case reports in the literature [11–13]. The largest case series of patients with AML and a FIP1L1-PDGFRA rearrangement consists of 5 patients, all of whom achieved complete molecular remission with imatinib [14]. All of the patients described had histories of peripheral eosinophilia, which prompted targeted testing for the FIP1L1-PDGFRA rearrangement, since this rearrangement is cryptic and cannot be detected by conventional karyotype. The more widespread adoption of gene panel approaches may reveal that this rearrangement is also present in hematologic neoplasms with more subtle eosinophil findings, such as in our case.

The importance of detecting the FIP1L1-PDGFRA rearrangement in hematopoetic neoplasms cannot be understated as this fusion protein is exquisitely sensitive to imatinib. In this case, our patient had exhausted all conventional therapy for myeloid sarcoma and, until the discovery of this rearrangement by high throughput sequencing, was imminently terminal. As the number of therapeutics targeting specific genetic alterations increases and the use of gene panel testing for somatic changes expands, this scenario of unanticipated findings dramatically affecting a patient’s clinical course is bound to be repeated to the benefit of cancer patients. Moreover, NGS based somatic sequencing is increasingly being used both to direct patients into clinical trials that target the specific molecular alterations found in their tumor and as biomarkers to associate clinical response to therapy with genetic profiles [15]. The clinical utility of this testing might be enhanced for rare cancers or atypical presentations where current evidence for guiding therapy is limited and genomic characterizations may assist with treatment decisions [16]. The case report presented here highlights the importance of broad molecular analysis in cancer, especially in cases that may display atypical features, either clinically or pathologically.
